# Modifier variants in metabolic pathways are associated with an increased penetrance of Leber’s Hereditary Optic Neuropathy

**DOI:** 10.1038/s41431-025-01860-7

**Published:** 2025-05-09

**Authors:** Eszter Sara Arany, Catarina Olimpio, Ida Paramonov, Rita Horvath

**Affiliations:** 1 Faculty of Medicine, Imperial College London, London, UK; 2 Department of Clinical Neurosciences, University of Cambridge, Cambridge, UK; 3 Centro Nacional de Análisis Genómico (CNAG), Barcelona, Spain

**Keywords:** Genetics research, Neurological disorders

## Abstract

Leber’s hereditary optic neuropathy (LHON) is a debilitating mitochondrial disease characterised by bilateral painless vision loss. Despite being the most prevalent mitochondrial disorder, the precise pathophysiological mechanisms underlying the penetrance of LHON remain poorly understood. Nuclear modifier genes have been long suspected to affect phenotype-severity, however, specific cellular pathways implicated in the disease penetrance have been only suggested recently. In recent years, autosomal recessive variants in nuclear genes involved in complex I function and metabolic pathways were recognised to cause a typical LHON phenotype. This was proposed as a new autosomal recessive disease mechanism for LHON (arLHON). The association between nuclear variants and the LHON phenotype makes the nuclear pathways disrupted in arLHON the strongest candidates to act as modifiers of mitochondrial LHON (mLHON). In this study we systematically investigated a large cohort of 23 symptomatic and 28 asymptomatic individuals carrying one of the three primary mitochondrial LHON variants. We identified several heterozygous pathogenic nuclear variants amongst the affected individuals that were consistently linked to metabolic and complex I related pathways, mirroring those disrupted in arLHON. Our findings are consistent with the presence of a second hit in specific biological pathways impairing ATP production. We propose that in addition to the primary mitochondrial variants, disruption in these nuclear-encoded pathways drives the clinical manifestation of LHON. Genes involved in the same pathways also emerge as exciting candidates for future association with arLHON. The present study deepens our understanding of LHON’s pathophysiology and provides a new framework for identifying novel disease-modifying targets.

## Introduction

Leber’s hereditary optic neuropathy (LHON) is the most common disease associated with pathogenic variants in the mitochondrial DNA (mtDNA) with an approximate prevalence of about 1 in 27,000–31,000 [1]. LHON is characterised by acute or subacute bilateral painless visual loss typically manifesting in the second or third decade of life, and is frequently accompanied by dyschromatopsia and central or centrocecal scotomas [2].

The majority of LHON cases, around 90–95%, are linked to variants in the mtDNA which are maternally inherited. In order of frequency, the mitochondrial form of LHON (mLHON) is most commonly associated with one of three pathogenic variants: m.11778 G > A (p.Arg340His) in *MT-ND4*, m.14484 T > C (p.Met64Val) in *MT-ND6*, and m.3460 G > A (p.Ala52Thr) in *MT-ND1* [2]. Additionally, there are thirty other rare variants in the mtDNA that have been associated with the disease [3].

The three primary mtDNA variants m.11778 G > A, m.14484 T > C and m.3460 G > A disrupt the structure of NADH dehydrogenase subunit 4, 6 and 1 of Complex I (CI), respectively. This leads to the dysfunction of CI of the mitochondrial electron transport chain resulting in decreased adenosine triphosphate (ATP) synthesis and the increased production of reactive oxygen species (ROS). The inherently increased energy demands of the retinal ganglion cells (RGCs) and the subsequent damage caused by accumulated ROS renders RGCs particularly vulnerable to declining levels of ATP. This cascade is thought to culminate in cellular death and axonal degeneration [2]. The only available treatment is the newly approved coenzyme Q10 analogue, idebenone, which can improve the prognosis of LHON. This drug facilitates the bypass of dysfunctional CI, thereby restoring ATP synthesis in RCGs [4].

Despite some successful interventions and the longstanding research of LHON pathogenesis since the first association with mitochondrial variants in the 1980s, the exact mechanisms of the disease are still unclear. Male predominance amongst affected patients is a characteristic hallmark of LHON with a male to female ratio of 3.2:1 for m.11778 G > A, 3.0:1 for m.14484 T > C, and 2.7:1 for m.3460 G > A [5]. The penetrance of LHON is, however, variable and incomplete, as only approximately 50% of men and 10% of women with a pathogenic variant show any significant symptom [6]. Remarkably, families possessing the same variants in a homoplasmic fashion also showed variable penetrance [6, 7], suggesting that the primary mitochondrial variants are necessary, but alone not sufficient to precipitate the disease.

Several factors were suggested to increase the likelihood of LHON penetrance. Tobacco and alcohol consumption, hormonal differences and vitamin deficiencies [5, 8] were historically thought to contribute to disease progression, however, the evidence for this is highly disputed [9].

Many genetic modifiers have also been previously investigated, including heteroplasmy [10], mitochondrial variants [11] and mtDNA haplogroups [12], without providing a convincing explanation.

There is an increasing body of evidence supporting the importance of polymorphic nuclear variants in modifying the pathogenicity of the primary genetic cause of rare diseases. Evidence for this was found in Huntington’s disease [13], maternally inherited non-syndromic sensorineural deafness [14], 22q11.2 deletion syndrome [15], cystic fibrosis [16], retinitis pigmentosa [17] and spinal muscular atrophy [18]. We have also shown in a rare mtDNA-related disease called reversible infantile respiratory chain deficiency, that digenic interaction of the homoplasmic m.14674 T > C *MT-TE* mutation and heterozygous nuclear variants in genes involved in mitochondrial translation contribute to the clinical phenotype [19].

Nuclear modifier variants exerting an influence on the LHON phenotype remain, however, poorly understood. X-linked variants received particular interest due to the male predominance in the condition. While some studies identified potential susceptibility loci on the X chromosome, including Xq25-27.2, suggesting a role for nuclear modifiers in disease expression, others did not confirm a consistent association or detect a specific modifying gene [7, 20, 21]. Investigations of several other nuclear genes related to mitochondrial biogenesis [22] or to mitochondrial function [7, 23] also failed to identify a variant of interest, except for *EPHX1* and *TP53*, which were linked to age of onset [24].

Remarkably, a recent study found association between pathogenic variants affecting CI and the phenotypic severity of LHON [25]. Reinforcing these findings, Blickhäuser et al. also demonstrated an association between a more severe mitochondrial phenotype, Leigh syndrome, and the co-occurrence of primary LHON variants with heterozygous mutations in CI subunit genes [26].

In recent years a number of autosomal recessive nuclear variants have been also linked to LHON (arLHON). Patients with arLHON (OMIM:619382) lack the primary pathogenic mitochondrial variants but possess autosomal recessive pathogenic variants and exhibit similar phenotype to mitochondrial LHON (mLHON). Beyond arLHON, other autosomal recessive optic neuropathies (arOAs) have also been reported to share overlapping features with LHON causing a LHON-like phenotype, and are often characterised by mitochondrial dysfunction and RGC degeneration. While these conditions have traditionally been categorized as distinct from LHON, recent studies suggest a continuum between arLHON and arOAs [27, 28]. This association makes the pathways affected in arLHON and arOAs the strongest candidates for mLHON modifiers. Several of these nuclear genes were associated with CI assembly and function (*NDUFS2* [29]*, DNAJC30* [30]*, NDUFA12* [31]*, TMEM126A* [32]), however, many were also linked to fatty acid and co-factor metabolism (*MCAT* [27]*, MECR* [33]) and other metabolic pathways (*ACO2* [34], *RTN4IP1* [28]). This further supports the idea that both CI related and metabolically related genes are likely candidates to cause an autosomal recessive form of LHON or act as LHON modifiers.

In this study we systematically investigated a cohort of patients carrying one of the three primary mitochondrial variants, and identified promising nuclear pathways that could be implicated in disease penetrance. We propose that a ‘second hit’ contributes to precipitating LHON in individuals carrying one of the three primary mitochondrial variants. Furthermore, we present additional evidence for the role of variants in CI related genes and metabolic enzymes in modifying the LHON phenotype.

## Materials and methods

### Participant recruitment

The RD-Connect Genome-Phenome Analysis Platform (GPAP) [35] is a user-friendly resource of genomic and phenotypic data of patients with rare disease and family members. GPAP facilitates diagnosis and gene discovery, and has been used as the primary analysis tool in a number of large European projects. It allows for registered members to reanalyse the genomic data from enroled participants maximising the chances of new diagnostic discovery and further understanding of disease mechanisms.

Genomic data from 2859 genomes and 22191 exomes visible to all registered and authorised users within this platform was filtered for the presence of one of the three most common LHON pathogenic mitochondrial variants (m.11778 G > A, m.14484 T > C and m.3460 G > A). We identified 51 participants fulfilling these criteria. As the RD-Connect GPAP also provides phenotype information, participants where then assigned an ‘affected’ (*n* = 23) or ‘unaffected’ status (*n* = 28) by an expert clinician. Unaffected participants were either healthy or had a phenotype not consistent with LHON. The full phenotypic description of unaffected individuals can be found in the Supplementary Information.

### Variant identification

We have identified rare variants in the selected cohort of 51 patients using the RD-Connect GPAP. Nuclear genes present in the mitochondrial related disorders v.8.7 Genomics England PanelApp panel were selected. Additionally, 27 further genes that were identified via a literature review and were implicated in LHON penetrance were added. The full list is available in the Supplementary Information. We then applied standard filtering criteria including moderate to high variant effect predictor (VEP) score (i.e. nonsense, splice site, frame-shift, in-frame and non-synonymous variants), gnomAD [36] allele frequency of <0.01 and a GPAP internal frequency <0.02. These threshold levels were selected to exclude common polymorphisms unlikely to play a major role in disease risk. gnomAD contains a broad population dataset representing a diverse set of healthy individuals. A stricter threshold (0.01) commonly used in rare disease research was applied to ensure the inclusion of rare variants only. GPAP RD-Connect is a rare disease-specific database, which already contains a higher proportion of individuals with rare genetic disorders. Therefore, a higher threshold (0.02) allowed for the inclusion of potentially pathogenic variants that may be overrepresented in a rare disease cohort. The aim of the higher set threshold value was to prevent excluding potentially biologically relevant variants.

Variant pathogenicity for each SNV was determined using the scoring system in the ACMG [37] and the ACGS-2024 variant interpretation guidelines [38]. Variants scoring at least 6 points were included in the pathogenic variant analysis.

### Computational analyses

Computational analyses and statistical tests were conducted in R (v4.4.1). Gene function assignment was extracted from the Mitocarta 3.0 [39] and the STRING databases [40]. Pathway analysis was performed using the ‘clusterProfiler’ package with the ontology database of the Kyoto Encyclopaedia of Genes and Genomes (KEGG) [41]. This method identifies relevant biological pathways by first mapping input gene sets to the pathways in the KEGG ontology database and then performing enrichment analysis to assess whether specific pathways are significantly overrepresented.

The test is based on the hypergeometric probability distribution that describes the likelihood of obtaining a specific number of positive outcomes in a finite population. Under this assumption the p value can be calculated as:

$$p=1-\sum \limits_{i=0}^{k-1}\frac{\left({M}\atop{i}\right)\left({N-M}\atop{n-i}\right)}{\left({N}\atop{n}\right)}$$

where:

*N* is the total number of genes in the background (all genes in KEGG)

*M* is the total number of genes associated with the pathway

*n* is the number of input genes

*k* is the number of input genes that overlap with the pathway.

To account for multiple hypothesis testing across all tested pathways, *p* values generated during this analysis were adjusted to control for false discovery rate. *P* values were adjusted using the Benjamini-Hochberg method and were considered significant with a value of <0.05.

## Results

We have searched a large database, the RD‐Connect Genome‐Phenome Analysis Platform (GPAP) [35] to identify individuals carrying one of the three primary mitochondrial variants. Analysis of a total of 2859 genomes and 22191 exomes identified 51 such individuals, all from different families. At the time of enrolment to GPAP 23 patients exhibited the LHON phenotype (affected cohort) and 28 patients did not show signs and symptoms consistent with LHON (unaffected cohort). This aligns with an estimated carrier frequency of 1 in 500 and an affected frequency of 1 in 1,100 individuals. These ratios differ from the globally recognized frequencies of primary pathogenic LHON variants, likely due to the GPAP database’s composition, which primarily includes individuals affected by rare diseases and their family members. However, the recently reported LHON primary mutation frequency of 1 in 800 in control Anglo-Saxon populations suggests some variability in carrier prevalence across different cohorts, potentially influenced by population structure and database composition [1].

The unaffected cohort consisted of 4 individuals showing no disease phenotype and 24 individuals affected by diseases other than LHON. The phenotypes included neuromuscular disorders, neurodevelopmental diseases and intellectual disability, neurodegenerative diseases and dementias, movement and extrapyramidal disorders, epilepsies and cancer. The complete phenotypic description of individuals can be found in Supplementary Table 1. There was male predominance in both groups (78% in the affected group, 61% in the unaffected group, p = 0.232, Fisher’s exact test). Data on age of onset, ethnicity and clinical severity were unavailable. Among the affected group, 10 individuals had m.3460 G > A, 11 had m.11778 G > A, and 3 had m.14484 T > C, whereas in the unaffected group, these numbers were 0, 9, and 20, respectively. Table 1 shows a detailed cohort comparison.

**Table 1:** Cohort characteristics.

	Affected	Unaffected
total participants	23	28
Female	5	11
Male	18	17
m.3460 G > A	10	0
m.11778 G > A	11	9
m.14484 T > C	3	20
Heteroplasmy level, mean (standard deviation)	94 (13.4)%	88 (31.5)%

### Rare variant analysis

**Fig. 1:**
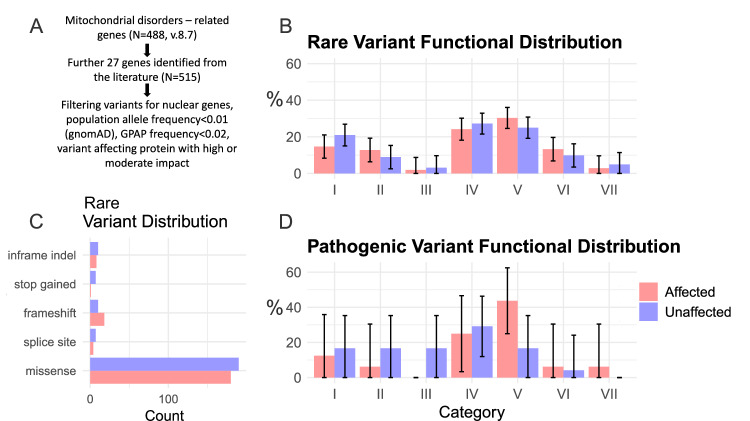
Graphical presentation of results. Analysis pipeline (**A**), functional distribution of rare variants based on Mitocarta 3.0 [39] (**B**), rare variant distribution by variant type (**C**), functional distribution of rare pathogenic variants based on Mitocarta 3.040 (**D**). Error bars represent the standard errors calculated assuming binomial distribution. Categories I-VII represent: mitochondrial complex assembly and function (I), mitochondrial transport and homoeostasis (II), mitochondrial fusion, fission and mitophagy (III), mitochondrial DNA and RNA processing (IV), metabolism (V), cellular structure and transport (VI) and other regulatory function (VII).

We have identified rare nuclear variants linked to mitochondrial function in each cohort resulting in 211 and 224 variants in the affected and unaffected group, respectively. To determine if the functional distribution of variants is different between the two groups, we categorised each gene into one of seven functional categories based on the Mitocarta 3.0 [39] and the STRING databases [40]: mitochondrial complex assembly and function, mitochondrial transport and homoeostasis, mitochondrial life cycle (fusion, fission, mitophagy), mitochondrial central dogma (DNA and RNA processing), metabolism, cellular structure and transport and other regulatory function. We observed no significant functional distribution differences between the cohorts (*p* = 0.212 chi-squared test). This is illustrated on Fig. 1B.

This result suggested no functional difference amongst the identified rare gene variants that could explain the phenotypic differences.

As CI-related modifiers have been previously linked to increased LHON penetrance [25], we further investigated if such modifiers are variably expressed in our cohort. CI-related genes were identified using the same databases as a subset of mitochondrial complex assembly and function related genes. We found that the variant burden affecting genes related to CI was not different between the two groups (*p* = 0.648, Fisher’s exact test): 48.3% and 42.5% of OXPHOS-related gene variants were linked to CI in the affected and unaffected cohort, respectively.

Given these results, we next hypothesised that modifier variants are likely to come from a subset of genes involved in specific pathways affected differently in probands and carriers.

### Analysis of preselected genes based on affected sample rate

To test this hypothesis, using the combined gene burden method, the affected sample rate for each previously identified gene was calculated in the affected and unaffected group. To identify key genes with the highest potential to explain differences between the two cohorts we further analysed a subgroup of genes with the highest difference in affected sample rate (*N* = 28, difference > 0.1). Conducting pathway analysis on this preselected gene list showed statistical significance for ‘Metabolism of cofactors and vitamins’ (*p* = 0.027) involving the genes *DHTKD1* and *DLAT*. Notably, these results are consistent with the findings of Cheng at al. [42] in a similar analysis of a different cohort.

### Pathogenic variants analysis

All previously identified rare variants were manually curated using the ACMG [37] and ACGS-2024 variant interpretation guidelines [38]. This identified N = 16 and N = 24 pathogenic or likely pathogenic variants in the affected and unaffected group, respectively, which have been included for further analysis. For simplicity, these variants are collectively referred to as pathogenic variants throughout the remainder of the text. Supplementary Table 3 contains further details on the variants.

The identified variants included a mix of missense and loss-of-function changes. Among the identified pathogenic variants several were predicted to have significant functional consequences, including nonsense variants disrupting essential catalytic domains and missense variants in highly conserved regions or with strong computational evidence for deleterious effects. Given the delicate pathways maintaining mitochondrial homoeostasis, these pathogenic variants could contribute to mitochondrial dysfunction even in a heterozygous state, with subtle impairments potentially influencing LHON penetrance.

Notably, there was a strong predominance of variants in genes involved in metabolic function (Category V) in the affected cohort (*N* = 7, 43.8%), which was not observed in the unaffected cohort (*N* = 4, 16.7%). This difference did, however, not reach the threshold of statistical significance, likely attributed to small sample size (*p* = 0.08, Fisher’s exact test). The results are illustrated on Fig. 1D.

Importantly, this analysis only showed the overall increased incidence of pathogenic variants in metabolic genes in the affected group without considering specific metabolic functions. Based on this trend, we then performed a more refined analysis to determine whether specific metabolic pathways are overrepresented amongst these pathogenic variants in the affected cohort. To achieve this, we conducted pathway analysis on the pathogenic variants in the affected and unaffected groups, respectively. This second analysis focused not only on the presence of metabolic genes but also on the specific pathways they are involved in, allowing for a more detailed characterisation of metabolic pathway enrichment in the affected cohort. We found that several metabolic pathways appeared statistically significant in the affected cohort, that were not identified in the unaffected cohort (Table 2).

**Table 2:** Statistically significant cellular pathways identified in pathway analysis of pathogenic variants.

Gene name	Category	Description of Pathway	Adjusted *p* value
**Affected Cohort**			
*DLAT/MDH2*	Metabolism	Citrate cycle (TCA cycle)	0.0026
		Pyruvate metabolism	0.0055
		Carbon metabolism	0.0271
*ACADM/ACADSB*	Metabolism	Fatty acid degradation	0.0051
		Valine, leucine and isoleucine degradation	0.0055
		Fatty acid metabolism	0.0072
**Unaffected Cohort**			
*FH/SDHD*	Metabolism	Citrate cycle (TCA cycle)	0.0143
*CPT2/SDHD/UQCRFS1*	Human Diseases	Diabetic cardiomyopathy	0.0365

The presented pathway *p* values were adjusted using Benjamini-Hochberg correction within the ClusterProfiler package to account for multiple pathway testing.

Notably, these pathways involved the genes *DLAT, MDH2, ACADSB*, and *ACADM*, which possessed variants with significant predicted functional consequences. Variants in *ACADSB* and *DLAT* were located at donor splice sites (with a SpliceAI score of 1 and 0.89, respectively), likely leading to aberrant splicing and premature protein truncation. Additionally, missense variants were identified within an evolutionarily highly conserved region in *ACADM* and adjacent to or directly overlapping with a key cofactor-binding region in *MDH2*.

In contrast to the rare variant analysis, investigating CI involvement amongst pathogenic variants revealed clear segregation between the cohorts. In the affected group, *N* = 2 OXPHOS-related pathogenic variants were found in *ACAD9* and *NDUFS1*, both implicated in CI function, whereas amongst the unaffected, *N* = 4 OXPHOS-related pathogenic variants were identified in *SDHD*, *UQCRFS1* and *APOPT1*, implicated in complex II, III and IV, respectively, but not in CI. Interestingly, these observations are consistent with the previously hypothesised involvement of pathogenic modifiers in CI-related genes in enhancing penetrance [25], however, given the small cohort size and a small number of pathogenic variants, the current results do not provide definitive evidence.

Our analysis did not identify any rare variants in the previously suggested *TP53* and *EPHX1* genes. Nor did we identify any relevant *OPA2* variants or other genes that lie on the X-chromosome and could eventually explain the bias between sexes.

## Discussion

LHON is the most common mitochondrial disorder affecting 1 in 27,000–31,000 patients. The care of individuals with LHON is complex and requires special considerations.

Providing patients with an expected prognosis is challenging due to the heterogeneous presentation of symptoms and potential fluctuations in visual loss. There is an emerging need for accurate genetic counselling for unaffected carriers to enable informed reproductive decision-making. However, this remains difficult due to the variable penetrance of the disease.

In this study we investigated a large cohort of patients possessing one of the three most common mitochondrial LHON variants, in order to identify potential nuclear variants and pathways implicated in precipitating the disease. Using various bioinformatics tools, we analysed the exome and genome sequencing data from 51 individuals carrying at least one of the pathogenic variants. Similar to previous findings [42], we identified differential expression of rare variants in the ‘co-factor and vitamin metabolism’ pathway, particularly involving the genes *DHTKD1* and *DLAT*, reinforcing the potential role of this pathway in modulating phenotypic differences between affected individuals and carriers.

The association of metabolic pathway dysfunction to phenotype enhancement is further reinforced by our pathogenic variant analysis. Remarkably, amongst the rare variants in the affected cohort a high proportion of pathogenic variants was found to be involved in metabolic functions (*ACAD9, ACADM, ACADSB, DLAT, NDUFS1, MDH2* and *SLC25A42*). This phenomenon was not observed in the unaffected group. Another interesting observation in our study was the clear clustering of pathogenic CI-related variants to the affected cohort. CI function disruption is thought to underlie the molecular cause of LHON, therefore, biologically, it seems plausible that a further hit to CI would increase an individual’s chances of developing the LHON phenotype, as it was also demonstrated recently [25]. As an alternative explanation, we cannot exclude that the pathogenic variants in genes encoding subunits of CII, CIII and CIV identified in unaffected individuals convey protection against the onset of LHON, conversely to those identified in CI subunits in affected individuals.

**Fig. 2:**
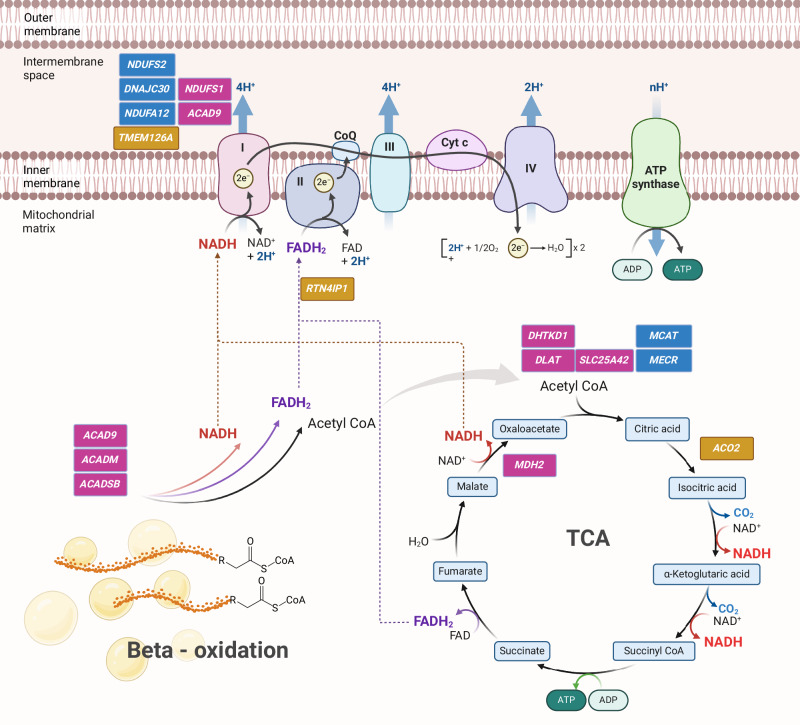
Illustration of the metabolic pathways affected by pathogenic variants in the affected cohort. The purple squares show the genes with pathogenic variants in the present study, the blue squares mark genes associated with arLHON, yellow squares indicate genes associated with arOA.

Placing the identified variants on a pathway and unifying the observations of this study can explain how a second metabolic insult could contribute to a stronger LHON phenotype. (Fig. 2).

Several of the genes identified in the affected cohort are directly involved in acetyl-CoA production. DHTKD1 is component of the 2-oxoadipate dehydrogenase complex (OADHC) [43] and participates in the rate limiting step of alpha-ketoadipate to glutaryl-CoA, which can eventually be degraded via β-oxidation to produce two acetyl-CoA molecules. DLAT, found in the inner mitochondrial membrane, forms part of the pyruvate dehydrogenase complex and catalyses the overall conversion of pyruvate to acetyl-CoA linking glycolysis and the TCA cycle [44]. *SLC25A42* encodes a mitochondrial transporter that transports coenzyme A (CoA) and adenosine 3’,5’-diphosphate across the inner mitochondrial membrane [45]. Notably, also *MCAT* and *MECR* (previously implicated in arLHON) play a key role in the biosynthesis of 2-oxoadipate dehydrogenase and pyruvate dehydrogenase, as well as other important TCA enzymes.

Acetyl-CoA is degraded in the TCA cycle to transfer electrons to the electron transport chain via NADH directly to CI. The protein encoded by *MDH2* catalyses the reversible oxidation of malate to oxaloacetate in the TCA cycle as well as plays a role in the malate-aspartate shuttle between mitochondria and cytosol [46]. As part of the same pathway, the arOA-related ACO2 catalyses the reversible conversion of citrate to isocitrate in the TCA cycle.

CI integrity is essential in receiving electrons from the TCA cycle and many arLHON genes’ function is linked to this complex. NDUFS1 forms a key subunit of CI localising to the inner mitochondrial membrane. The subunit’s main function is directly transferring electrons from NADH to ubiquinone and down the respiratory chain [47]. ACAD9 forms part of the MCIA complex and participates in aiding assembly of CI. ACAD9 is also a member of the acyl-CoA dehydrogenase family specifically contributing to the catalysis of the first step in beta oxidation of palmitoyl-CoA and long-chain unsaturated substrates, similar to ACADM and ACADSB explained below [48].

The electron transport chain’s second entry point is via complex III. ACADM and ACADSB catalyse the first step of mitochondrial medium-chain and short chain fatty acid oxidation, respectively, essential for the formation of acyl-CoA [49]. Remarkably, during this process electron transfer flavoprotein also receives and transfers electrons to the respiratory chain via coenzyme Q10 entering the chain at complex III, which aligns with the role in coenzyme Q biosynthesis of the arOA-related RTN4IP1 [50]. Notably, idebenone, the only approved treatment to slow the progression of LHON, is a coenzyme Q10 analogue that enhances oxidative phosphorylation by bypassing Complex I. Therefore, any additional impairment in this pathway could plausibly exacerbate the severity of the phenotype.

Two genes have been identified in the unaffected cohort found on the same pathways: *FH*, which forms part of the TCA cycle and *CPT2* involved in the beta-oxidation of long-chain fatty acids. We speculate that these unaffected individuals harbouring pathogenic variants in these genes are at increased risk of developing the phenotype in the future.

The observations in this study, that the above-described genes form part of a functional pathway in energy production further support the hypothesis that modifier variants are enriched in specific metabolic biological pathways impairing ATP production. These pathways are likely to coincide with those implicated in the pathophysiology of arLHON. The clinical severity of LHON is likely influenced by multiple genes and pathways that remain to be further elucidated. Based on our findings, we hypothesise that a second insult within the metabolic pathway—either upstream of the electron transport chain or directly affecting CI—further impairs oxidative phosphorylation, resulting in an even greater reduction in ATP production in the already vulnerable RGCs. Subsequently, this ‘extra’ hit on energy production can lead to the exhaustion of the cellular compensatory mechanisms resulting in an individual experiencing the clinical symptoms.

Our study has several limitations. The age of onset for the clinical manifestation of LHON is highly variable and it cannot be excluded that some of the patients included in the study will develop the phenotype in the future. Several important demographic details were also missing from our data including lifestyle differences and ethnicity. Genetic ancestry can influence the prevalence of rare pathogenic variants, therefore, it is possible that differences in ancestry between affected and unaffected individuals contribute to the observed variant enrichment, potentially acting as a confounding factor in the analysis. Additionally, due to the small cohort size, the three LHON mtDNA variants were analysed collectively without consideration of potential variant-specific modifiers. Our cohort exhibited variability in the penetrance of the three primary mitochondrial LHON variants. Similar findings have been reported in previous studies [1], with m.14484 T > C more frequently observed in asymptomatic carriers, while m.3460 G > A is less commonly identified. These findings suggest inherent differences in the pathogenic potential of each variant, which are likely further influenced by nuclear or environmental modifiers. A variant-specific modifier analysis in a larger cohort could provide further insights into these disparities. Also, the unaffected cohort included individuals who were affected by other non-LHON phenotypes, which may have had an impact on identified variants. Certain conditions can trigger metabolic adaptations and mitochondrial compensatory mechanisms, and could theoretically influence mitochondrial resilience and modify disease severity. As, however, the observed phenotypes in the unaffected group were highly heterogeneous and not consistent with a metabolic disorder the likelihood of this is small. Furthermore, our study only investigated the role of rare variants as potential LHON modifiers. Future studies with larger cohorts could focus on eliciting the role of more common variants, potentially utilising the genome wide association study model. The conclusions of the present study could be further strengthened by detailed functional studies or segregation analysis in single families.

The results presented here provide a valuable foundation for future studies involving larger cohorts or model organisms to validate and expand upon these findings, potentially offering new insights into the molecular mechanisms driving the LHON phenotype. Exploring these pathways cannot only identify more mLHON modifiers but also highlight new candidate genes for arLHON. Further elucidating the mechanisms of potential second metabolic insults in mLHON could aid the development of new therapies aimed at restoring metabolic function and enhancing cellular resilience, potentially drawing on strategies similar to those employed in the treatment of rare inborn errors of metabolism.

## Supplementary information

SI Table 1: Detailed phenotype of individuals in the unaffected cohort

SI Table 2: All genes searched

SI Table 3: Pathogenic and likely pathogenic variants

## Data Availability

Data is available from the corresponding author upon reasonable request.
